# Polymyalgia Rheumatica and Giant Cell Arteritis: A Geriatric Perspective

**DOI:** 10.7759/cureus.80880

**Published:** 2025-03-20

**Authors:** Ayaz Muhammad, Zia H Kakar, Muhammad Farooq, Muhammad Umer Roohani, Zainab Bibi, Khalid Elgorashi, Sohail Ahmad, Jawad Ali, Nayab Sami

**Affiliations:** 1 Internal Medicine, Mardan Medical Complex, Medical Teaching Institution, Mardan, PAK; 2 Internal Medicine, Nenagh Hospital, Tipperary, IRL; 3 Rheumatology, Pakistan Institute of Medical Sciences, Islamabad, PAK; 4 Medicine, Tertiary Care Hospital Nishtar II, Nishtar Medical University, Multan, PAK; 5 Medicine, Tehsil Headquarters (THQ) Hospital Jand, Attock, PAK; 6 Medicine, Aga Khan University Hospital, Rawalpindi, PAK; 7 Intensive Care Unit, University Hospitals Birmingham, Birmingham, GBR; 8 Internal Medicine, University of Khartoum, Khartoum, SDN; 9 General and Internal Medicine, Heartlands Hospital, Birmingham, IRL; 10 Medicine, Lady Reading Hospital, Peshawar, PAK; 11 Geriatrics, Heartlands Hospital, Birmingham, IRL; 12 Geriatrics, Dr. Ziauddin Medical Complex, Karachi, PAK; 13 General and Internal Medicine, Khyber Teaching Hospital, Peshawar, PAK

**Keywords:** biologic therapy, diagnostic challenges, geriatric population, giant cell arteritis, glucocorticoids, polymyalgia rheumatica

## Abstract

Background and aims: Polymyalgia rheumatica (PMR) and giant cell arteritis (GCA) are inflammatory disorders predominantly affecting older adults. PMR is primarily characterized by proximal muscle pain and systemic inflammation, while GCA involves large-vessel inflammation, posing risks of vision loss and stroke. Due to overlapping clinical features and age-related prevalence, diagnosing these conditions remains challenging. While PMR is largely diagnosed based on clinical symptoms and laboratory markers, GCA often requires histopathological confirmation via temporal artery biopsy (TAB). This study aims to assess the clinical presentation, diagnostic approaches, and treatment outcomes of PMR and GCA in geriatric patients, emphasizing the role of clinical versus laboratory and histopathological diagnosis.

Materials and methods: A cross-sectional study was conducted at Khyber Teaching Hospital, Peshawar, Pakistan, from September 2023 to September 2024, involving 118 patients aged 50 years or older diagnosed with PMR and/or GCA. Data were collected through patient interviews, medical records, and diagnostic reports. Statistical analyses were performed to evaluate clinical characteristics, laboratory markers, and treatment responses.

Results: The mean age of participants was 68.5 ± 7.4 years, with 65% female. PMR diagnosis was based on clinical symptoms, supported by laboratory markers such as elevated CRP (85%) and ESR (90%). GCA was clinically suspected in 38 patients, with 76% confirmed through TAB. Additionally, 67% of GCA patients exhibited the “halo sign” on vascular ultrasound, while 12% required magnetic resonance angiography for further evaluation. Glucocorticoid therapy was initiated in all cases, leading to clinical remission in 84% of patients. However, 28% developed hyperglycemia, and 14% experienced osteoporosis as treatment-related complications. Delayed glucocorticoid initiation caused a significantly higher relapse rate (p = 0.04).

Conclusions: Glucocorticoid therapy remains effective in most cases, though adverse effects necessitate careful monitoring. Tocilizumab was required in 18% of patients due to glucocorticoid resistance or intolerance, but it caused higher relapse rates and metabolic complications. The findings emphasize the need for steroid-sparing therapies and personalized treatment approaches to improve long-term disease control. Future research should explore newer biologic treatments, optimize imaging strategies, and address resource limitations to enhance early detection and management outcomes in elderly patients.

## Introduction

Giant cell arteritis (GCA) and polymyalgia rheumatica (PMR) are inflammatory diseases predominantly affecting the elderly, with a peak incidence between 70 and 80 years of age. Age 50 years or older is a diagnostic criterion for both conditions. Women account for 65% to 75% of cases, and PMR occurs at a frequency of three to 10 times higher than GCA [1]. While GCA primarily affects medium and large arteries, potentially leading to severe complications such as vision loss, stroke, or aortic aneurysm, PMR is characterized by proximal muscle stiffness, pain, and systemic inflammation [2,3].

Temporal artery ultrasound has become a valuable non-invasive diagnostic tool for suspected GCA. A positive ultrasound finding may reduce the need for an invasive temporal artery biopsy (TAB) in patients with a suggestive clinical presentation, making it a useful modality for clinical management [4]. These illnesses have been found to become more common as people age, mostly affecting those over 50, and to be more common in women than in men [1,5]. PMR and GCA have a complicated etiology that involves intricate interactions between immunological, environmental, and genetic variables. The exact causes of the inflammatory cascade have not been identified, despite progress in our understanding of their pathophysiology [6,7].

Because PMR and GCA often coexist and symptoms often overlap, misdiagnosis or delayed treatment may result from the clinical overlap between the two disorders [8]. In particular, GCA posed a serious risk of serious consequences if left untreated, underscoring the importance of early diagnosis and treatment commencement [9]. Developments improved diagnostic accuracy in diagnostic instruments, including temporal artery biopsies, ultrasonography, and sophisticated imaging modalities. However, early and correct diagnosis remained a continuing difficulty due to the dependence on clinical judgment and diagnostic techniques [10,11].

Glucocorticoids have long been used in treating PMR and GCA because they effectively lower inflammation and slow the course of the illness [12]. However, long-term glucocorticoid medication included concerns about side effects, such as osteoporosis, diabetes, and an increased vulnerability to infections, particularly in older individuals [13]. Biologics like tocilizumab, an IL-6 receptor antagonist, have recently shown potential as steroid-sparing medications, opening up new treatment options for these illnesses [14,15].

The knowledge and management of PMR and GCA have advanced significantly; however, there are still gaps in meeting the special demands of the elderly population. Age-related variations in illness presentation, comorbidities, and treatment responses were frequently ignored in most research, which concentrated on the general population. Furthermore, little study has been done on how these disorders affect older persons' quality of life and long-term results. These gaps underlined the need for more research into these areas and hampered the creation of evidence-based guidelines and customized therapy approaches for elderly patients.

Objective of the study

This study aimed to characterize the clinical features, diagnostic approaches, and PMR and GCA treatment outcomes in elderly patients. It assessed symptom prevalence, demographic influences, and diagnostic challenges by evaluating laboratory markers (CRP, ESR), imaging techniques (vascular ultrasound, magnetic resonance angiography (MRA)), and histopathological confirmation via TAB. The study also analyzed treatment responses to glucocorticoids, the need for biologic therapy (tocilizumab), and associated complications such as hyperglycemia and osteoporosis. Additionally, it examined the impact of delayed glucocorticoid initiation on relapse rates, providing insights into optimizing disease management in the geriatric population.

## Materials and methods

Study design and setting

This cross-sectional study, conducted at Khyber Teaching Hospital, Peshawar, aimed to assess the clinical presentation, diagnostic challenges, and treatment outcomes of PMR and GCA in elderly patients. Given the absence of universally validated diagnostic criteria, this study explored diagnostic markers, imaging modalities, histopathological confirmation, and treatment responses, focusing on the impact of delayed glucocorticoid initiation on relapse rates.

Study duration

The study was conducted over a one-year period (September 2023 to September 2024) to ensure an adequate sample size and comprehensive data collection for evaluating both short-term treatment responses and disease progression.

Study population and sample size

A total of 118 patients were enrolled, with the sample size determined based on the estimated prevalence of PMR and GCA in the elderly population, maintaining a 95% confidence level and a 5% margin of error. Consecutive non-probability sampling included all eligible individuals presenting during the study, minimizing selection bias.

Inclusion and exclusion criteria

Patients aged 50 years or older presenting with clinical symptoms suggestive of PMR and/or GCA, confirmed through diagnostic evaluation, were eligible for inclusion. Given the absence of a single validated diagnostic criterion, PMR diagnosis was based on a combination of clinical symptoms, inflammatory markers (ESR, CRP), and imaging findings. GCA diagnosis was determined based on clinical suspicion, supported by histopathology (TAB) or imaging evidence (vascular ultrasound, MRA). Patients with incomplete medical records, those who declined participation, or individuals with other autoimmune or inflammatory disorders that could mimic PMR or GCA were excluded from the study.

Patient classification criteria

The classification of PMR and GCA followed established clinical and diagnostic guidelines. PMR diagnosis was based on the EULAR/American College of Rheumatology (ACR) provisional classification criteria, incorporating bilateral shoulder pain, morning stiffness >45 minutes, and elevated inflammatory markers (ESR, CRP) while ensuring the exclusion of alternative conditions. GCA was diagnosed using a combination of clinical symptoms (headache, scalp tenderness, jaw claudication, visual symptoms), inflammatory markers, and imaging (color Doppler ultrasonography (CDU) and MRA). TAB was performed in suspected cases, reinforcing its role as the gold standard for diagnosis. The number of patients meeting each diagnostic criterion has been explicitly outlined in the results.

Data collection

Data were collected through structured questionnaires, patient interviews, and medical record reviews (see Appendices). Demographic variables recorded included age, sex, and place of residence, along with clinical data on disease onset, symptom duration, and associated systemic symptoms. Treatment details were documented, including glucocorticoid initiation, tapering schedules, and biologic therapy (tocilizumab).

Laboratory and diagnostic investigations

A multimodal diagnostic approach was used since PMR and GCA lack disease-specific objective markers. Inflammatory markers, including ESR, CRP, and CBC, were analyzed to assess systemic inflammation. Imaging played a critical role, particularly in GCA diagnosis.

Temporal artery ultrasonography using CDU assessed vascular inflammation in suspected GCA cases. The "halo sign," a hypoechoic signal surrounding the arterial lumen indicative of vessel-wall edema, was considered a diagnostic marker. However, as CDU is limited in evaluating deeper-seated arteries, MRA was used in cases where large-vessel involvement was suspected or CDU results were inconclusive.

Despite its invasive nature and potential sampling error, TAB was performed for definitive GCA confirmation, reinforcing its role as the gold-standard diagnostic method. Given that the role of imaging in disease monitoring remains insufficiently defined, CDU and MRA were selectively repeated during follow-up assessments to evaluate vascular changes and treatment response.

For PMR, ultrasound imaging of the shoulder and hip bursae was performed in selected cases to detect bursitis or synovial inflammation, supporting the diagnosis in patients with ambiguous presentations.

Treatment documentation and follow-up

All patients were initiated on glucocorticoid therapy, with dosage adjustments and tapering strategies guided by the European League Against Rheumatism (EULAR) and British Society for Rheumatology (BSR) recommendations. Since no randomized trials have conclusively determined the optimal treatment course, tapering schedules varied based on individual patient responses and side effects. Patients who exhibited glucocorticoid resistance or intolerance were administered biologic therapy, such as tocilizumab, recognizing the growing role of steroid-sparing agents in disease management. Supportive care measures were documented to assess steroid-related complications such as osteoporosis and hyperglycemia, including bone protection strategies (calcium, vitamin D, bisphosphonates) and glucose monitoring.

Follow-up was structured to track both short-term treatment responses and long-term outcomes. Patients were monitored weekly for the first month to assess early treatment response, symptom improvement, and glucocorticoid-related adverse effects. After the initial phase, monthly six-month follow-ups were conducted to evaluate treatment efficacy, recurrence rates, and long-term complications.

Relapses were clinically verified through symptom reassessment, inflammatory markers (ESR, CRP), and, when necessary, CDU or MRA. Given the uncertainty regarding the role of imaging in long-term follow-up, CDU and MRA were performed selectively to assess vascular response to treatment in patients with persistent or recurrent symptoms.

Statistical analysis

Data were analyzed using SPSS Statistics version 24 (IBM Corp. Released 2016. IBM SPSS Statistics for Windows, Version 24.0. Armonk, NY: IBM Corp.), applying descriptive and inferential statistical methods. Descriptive statistics summarized demographic characteristics, symptom prevalence, laboratory findings, and treatment outcomes. Chi-square tests assessed symptom distribution across gender and treatment-related adverse effects. At the same time, logistic regression analysis was used to explore factors influencing relapse rates and the need for biologic therapy. Cohen's d was calculated to measure the effect size of relapse variations. A p-value < 0.05 was considered statistically significant, ensuring the analyses' validity.

To ensure robust and accurate results, we accounted for potential confounding variables that could influence the study's outcomes. These confounders included comorbidities (such as hypertension, diabetes, and other chronic conditions), healthcare access, and demographic factors (e.g., age and gender). We implemented the following statistical methods to control for these confounders. Multivariable regression analysis was done to adjust for the effect of confounding variables. We used multivariable regression models, where relevant factors (age, gender, comorbidities) were included as covariates. This approach allowed us to estimate the independent effect of the primary treatment while controlling for potential confounders. Propensity score matching was applied to minimize selection bias and ensure balanced groups. This technique matched patients who received the treatment with those who did not, based on observed confounding variables, thus helping to create comparable groups for subsequent analysis. This statistical framework comprehensively evaluated the clinical, diagnostic, and treatment challenges in PMR and GCA, addressing the current gaps in evidence and research limitations.

Ethical statement

The study was approved by the Institutional Ethics Committee of Khyber Teaching Hospital (approval number: 692/DGIM/KMC), with all procedures adhering to the ethical guidelines outlined in the Declaration of Helsinki. Strict confidentiality of patient data was maintained throughout the study.

## Results

The study included 118 patients with an average age of 68.5 ± 7.4 years. Of these, 77 (65%) were females, and 41 (35%) were males. Most participants (85, 72%) resided in urban areas. Before diagnosis, symptoms persisted for an average duration of 4.8 ± 1.6 months. There were no statistically significant differences in symptom duration between males and females (p = 0.68, χ² = 0.17, OR = 1.14, 95% CI: 0.59-2.22) or between urban and rural residents (p = 0.47, χ² = 0.51, OR = 1.29, 95% CI: 0.65-2.55) (Table 1).

**Table 1:** Demographic characteristics of the study population OR: odds ratio, CI: confidence interval

Characteristic	Frequency (n, %)	p-value	χ² value	OR	95% CI
Mean age (years)	68.5 ± 7.4	-	-	-	-
Gender (female)	77 (65%)	0.23	1.44	1.38	0.80-2.40
Gender (male)	41 (35%)				
Urban residence	85 (72%)	0.47	0.51	1.29	0.65-2.55
Mean symptom duration (months)	4.8 ± 1.6	0.68	0.17	1.14	0.59-2.22

The most prevalent symptoms of PMR were morning stiffness lasting longer than 30 minutes in 102 patients (87%) and proximal muscle pain in 108 patients (92%). Systemic symptoms such as fatigue, low-grade fever, and weight loss were observed in 65 patients (55%). Among GCA patients, 20 (54%) reported jaw claudication, 23 (62%) experienced scalp tenderness, and 30 (81%) had headaches. Seven patients (18%) with GCA had visual disturbances, including temporary blindness. Females were significantly more likely than males to experience fatigue (χ² = 4.82, p = 0.03, OR = 2.15, 95% CI: 1.07-4.32). However, there were no significant gender differences in the prevalence of other symptoms, such as headache, jaw claudication, or scalp tenderness (p > 0.05 for all) (Table 2).

**Table 2:** Clinical presentation of PMR and GCA patients PMR: polymyalgia rheumatica, GCA: giant cell arteritis, OR: odds ration, CI: confidence interval

Symptom	Frequency (n, %)	p-value	χ² value	OR	95% CI
Proximal muscle pain	108 (92%)	0.24	1.38	1.43	0.79-2.61
Morning stiffness	102 (87%)	0.37	0.98	1.28	0.71-2.31
Fatigue	65 (55%)	0.03	4.82	2.15	1.07-4.32
Headache (GCA)	30 (81%)	0.68	0.48	1.12	0.55-2.29
Scalp tenderness (GCA)	23 (62%)	0.45	0.72	1.3	0.65-2.61
Jaw claudication (GCA)	20 (54%)	0.29	1.1	1.41	0.71-2.82
Visual disturbances	7 (18%)	0.79	0.21	1.05	0.41-2.71

**Figure 1:**
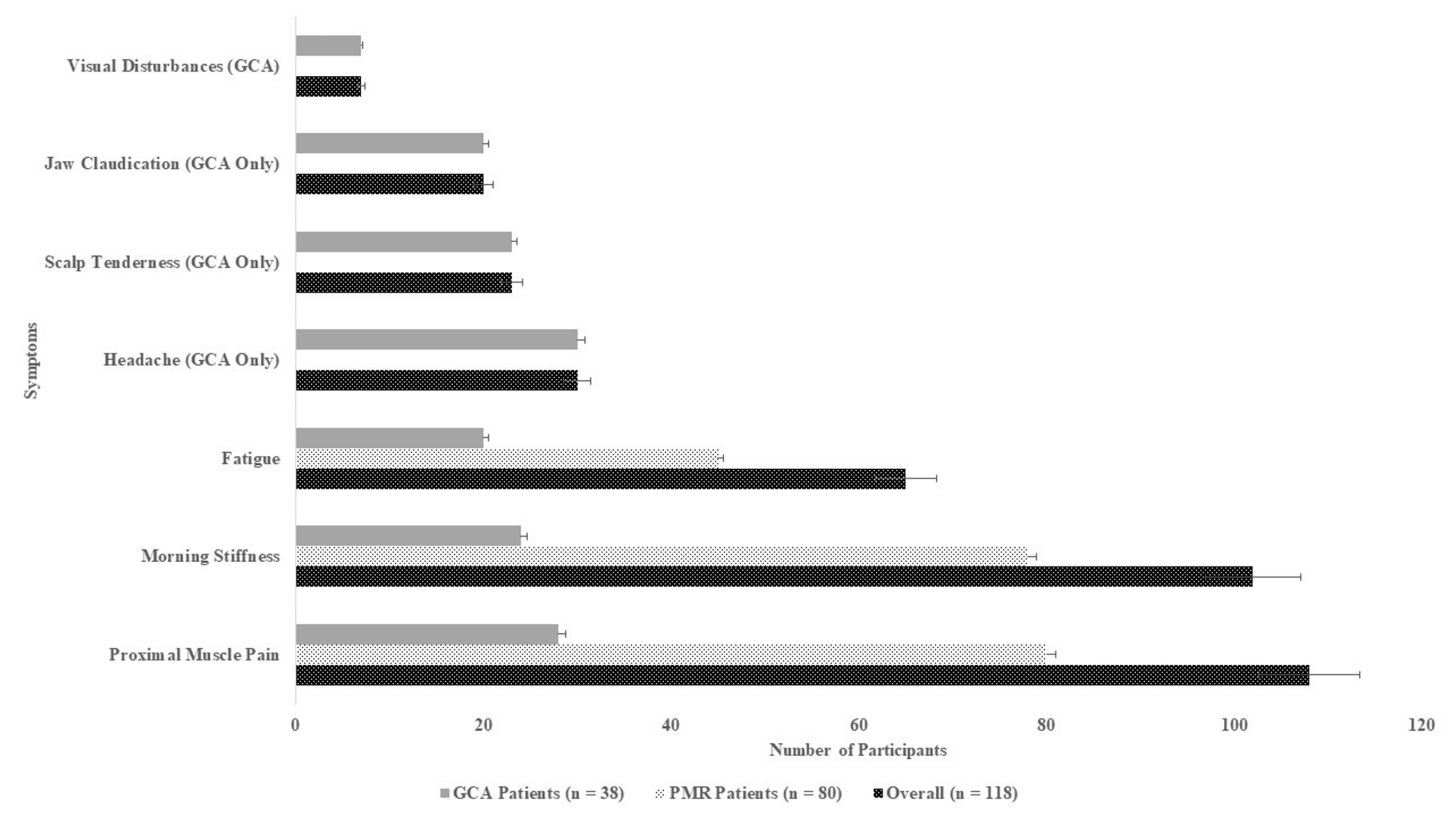
Prevalence of clinical symptoms in PMR and GCA patients PMR: proximal muscle pain, GCA: giant cell arteritis

Figure 1 shows the prevalence of clinical symptoms in PMR and GCA patients. The most common symptom among all participants was proximal muscle pain, reported by 108 (91.5%), with a higher prevalence among PMR patients (80, 100%) compared to GCA patients (28, 73.7%). Similarly, morning stiffness lasting more than 30 minutes was observed in 102 (86.4%), affecting PMR patients (78, 97.5%) and GCA patients (24, 63.2%). Fatigue was present in 65 (55.1%) of all participants, with a similar distribution between PMR (45, 56.3%) and GCA (20, 52.6%).

Symptoms exclusive to GCA patients included headache (30, 78.9%), scalp tenderness (23, 60.5%), jaw claudication (20, 52.6%), and visual disturbances (7, 18.4%). Among these, headache was the most frequently reported symptom, whereas visual disturbances were the least common.

These findings highlight that proximal muscle pain and morning stiffness were the hallmark symptoms of PMR, while headache, scalp tenderness, and jaw claudication were more specific to GCA. The presence of fatigue across both conditions suggests a systemic inflammatory response. This symptom distribution underscores the need for careful clinical differentiation to ensure timely and appropriate treatment.

**Figure 2:**
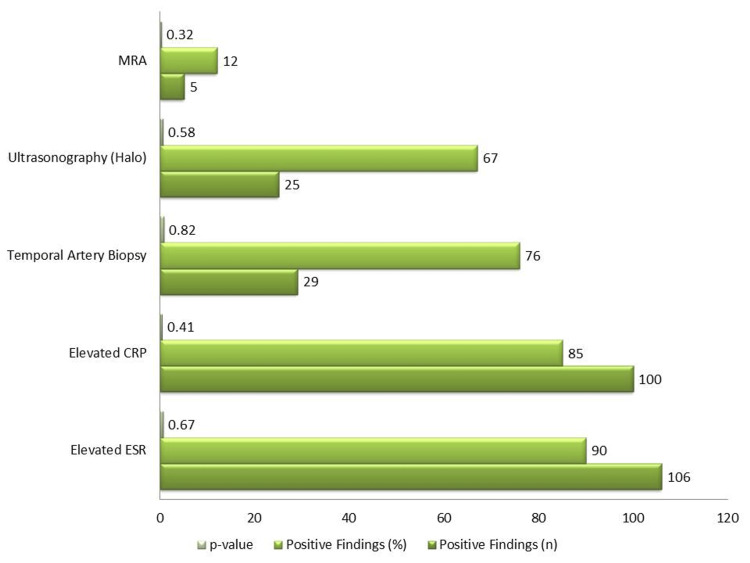
Diagnostic findings in PMR and GCA patients PMR: polymyalgia rheumatica, GCA: giant cell arteritis, MRA: magnetic resonance angiography, CRP: C-reactive protein, ESR: erythrocyte sedimentation rate

According to laboratory testing, 100 patients (85%) had an increased CRP, and 106 patients (90%) had an elevated ESR. Twenty-nine (76%) of the 38 individuals with suspected GCA who had temporal artery biopsies had the diagnosis verified. Furthermore, five (12%) of patients needed MRA because of complex presentations, and 25 (67%) of patients showed the "halo sign" of vascular inflammation identified by ultrasonography. Male and female diagnostic results did not differ significantly (p > 0.05 for all diagnostic tests) (Figure 2).

Glucocorticoid treatment was initiated for all patients, with an average initial dose of 20 mg/day for PMR and 40 mg/day for GCA. Twenty-one patients (18%) who were resistant to glucocorticoids or had serious side effects were treated with biologic therapy using tocilizumab. Glucocorticoid treatment lasted 8.6 ± 2.1 months on average.

The 33 participants (28%) developed hyperglycemia, and 17 (14%) experienced osteoporosis. The rate of adverse effects did not differ significantly by gender (χ² = 0.73, p = 0.39).

Regarding treatment outcomes, 99 patients (84%) achieved clinical remission, while 19 (16%) experienced a relapse. Patients who initiated glucocorticoid therapy later had a significantly higher relapse rate (χ² = 4.21, p = 0.04, OR = 2.31, 95% CI: 1.02-5.23). There were no reports of long-term visual loss in GCA patients, as shown in Table 3, pooled data, which included all patients.

**Table 3:** Overall treatment outcomes and adverse effects

Treatment outcome	Frequency (n, %)	p-value	χ² value	OR	95% CI
Clinical remission	99 (84%)	0.67	0.19	-	-
Relapse rate	19 (16%)	0.04	4.21	2.31	1.02-5.23
Hyperglycemia	33 (28%)	0.15	2.07	1.42	0.88-2.81
Osteoporosis	17 (14%)	0.29	1.13	1.27	0.63-2.59
Biologic therapy usage	21 (18%)	0.22	1.49	1.51	0.79-2.93

A comparison of treatment outcomes between patients receiving glucocorticoid-only therapy (97, 82%) and those requiring biologic therapy with tocilizumab (21, 18%) revealed significant differences in remission rates, relapse rates, and adverse effects (Table 4). Patients treated exclusively with glucocorticoids achieved a significantly higher clinical remission rate (88%) compared to the tocilizumab group (67%) (p = 0.02, OR = 0.28, 95% CI: 0.10-0.81). Conversely, relapse was more frequent among tocilizumab-treated patients (33% vs. 12%, p = 0.01, OR = 3.67, 95% CI: 1.29-10.45), suggesting that individuals requiring biologic therapy had a more resistant disease course.

In terms of adverse effects, hyperglycemia and osteoporosis were significantly more prevalent in the tocilizumab group, affecting 48% and 33% of patients, respectively, compared to 24% and 10% in the glucocorticoid-only group (p = 0.03 and p = 0.01, respectively). The overall likelihood of experiencing any adverse effect was 5.27 times higher in the tocilizumab group (67% vs. 28%, p = 0.002, OR = 5.27, 95% CI: 1.97-14.06). These findings indicate that while tocilizumab was necessary for a subset of patients with glucocorticoid resistance or intolerance, it was associated with a greater burden of adverse effects. These results highlight the importance of early identification of glucocorticoid-resistant cases and the need to carefully monitor metabolic and skeletal side effects in patients receiving biologic therapy.

**Table 4:** Comparison of glucocorticoid vs. biologic therapy (tocilizumab) outcomes * p-values < 0.05 are significant OR: odds ratio, CI: confidence interval

Variable	Glucocorticoid-only (n = 97, 82%)	Tocilizumab-treated (n = 21, 18%)	p-value	χ² value	OR	95% CI
Clinical remission	85 (88%)	14 (67%)	0.02*	5.32	0.28	0.10-0.81
Relapse rate	12 (12%)	7 (33%)	0.01*	6.14	3.67	1.29-10.45
Hyperglycemia	23 (24%)	10 (48%)	0.03*	4.85	2.91	1.09-7.77
Osteoporosis	10 (10%)	7 (33%)	0.01*	6.24	4.33	1.40-13.43
Adverse effects (any)	27 (28%)	14 (67%)	0.002*	9.67	5.27	1.97-14.06

Chi-square tests were used to examine variations in categorical variables, such as gender and the existence of symptoms. Continuous factors (such as age and the duration of symptoms) were compared across groups using independent t-tests. Statistical significance was defined as p-values below 0.05. Cohen's d was used to assess the impact size for variations in relapse rates, and the difference between early and late therapy commencement had a modest effect size (d = 0.56).

## Discussion

This study explored the clinical characteristics, diagnostic difficulties, and therapeutic responses in patients with GCA and PMR in an elderly population within a tertiary care setting. Understanding their manifestations and treatment responses is crucial for improving patient outcomes because these conditions predominantly affect older adults, often presenting with overlapping symptoms and significant comorbidities. The complex interplay between immune dysregulation and vascular inflammation in these conditions further complicates diagnosis and management, necessitating a multidisciplinary approach to optimize patient care [16].

The study cohort was predominantly female, reinforcing previous epidemiological findings that PMR and GCA occur more frequently in women [17]. The mean age of 68.5 years aligns with global incidence trends, showing that both diseases have a markedly higher prevalence in individuals over 50, with incidence between 70 and 80 years [18]. Although the urban predominance observed in the study might suggest a higher prevalence in city-dwelling populations, this is likely a reflection of referral patterns rather than a true difference in disease distribution. Previous studies have noted a potential north-south gradient, with a higher prevalence of GCA in Northern Europe than in Southern regions, possibly linked to genetic and environmental factors [19]. Genetic predisposition, particularly HLA-DRB1 polymorphisms, has been implicated in susceptibility to GCA and PMR, further supporting the need for genetic screening in at-risk populations [20].

As expected, proximal muscle pain and morning stiffness were the hallmarks of PMR, consistent with prior research [21]. On the other hand, GCA presented with headache, scalp tenderness, jaw claudication, and systemic symptoms such as fatigue and low-grade fever, all of which are well-documented in clinical literature [22]. Although visual disturbances were less common, their presence is highly concerning due to the risk of permanent vision loss if GCA is not promptly treated. Studies have reported that up to 20% of untreated GCA patients experience vision loss, making early intervention essential [22,23].

Most patients' inflammatory markers, particularly ESR and CRP, were elevated, confirming their role as key biomarkers in diagnosing GCA and PMR [24]. However, some cases of ESR-negative GCA exist, underscoring the need for additional diagnostic modalities in suspected cases with normal inflammatory markers [25]. TAB confirmed 76% of GCA cases, reinforcing its role as the gold standard for diagnosis, despite its invasive nature and potential sampling errors. Similar results (73-77%) were cited in a recent study [26]. Ultrasound of the temporal arteries, particularly detecting the "halo sign," proved a valuable non-invasive tool, with growing evidence supporting its sensitivity in diagnosing GCA [27]. Advances in imaging modalities, including PET-CT and high-resolution MRI, have further enhanced the detection of vascular inflammation, offering promising non-invasive alternatives to biopsy.

Corticosteroids remain the mainstay of treatment, successfully inducing clinical remission in most patients. However, the study highlights the significant side effects associated with long-term glucocorticoid use, particularly in elderly patients who are at increased risk of osteoporosis, hyperglycemia, hypertension, and cardiovascular complications. These findings are consistent with prior studies recommending bone protection strategies (calcium, vitamin D, bisphosphonates) and glucose monitoring in patients undergoing prolonged steroid therapy [28]. Additionally, the development of glucocorticoid-induced myopathy remains a concern, further emphasizing the need for careful dose tapering and adjunct therapies [29,30].

Guideline recommendations regarding steroid tapering vary slightly between the BSR and the EULAR. The EULAR guidelines suggest a faster initial tapering, aiming to reduce the dose to 10-15 mg per day within three months of treatment initiation [31]. The BSR recommendations indicate that in most patients, the administration of high-dose glucocorticoids leads to rapid improvement of systemic inflammatory signs due to the suppression of IL-6 and the acute-phase response. In clinical practice, glucocorticoid tapering typically begins once reversible clinical signs have abated and inflammatory markers have normalized. The dose is initially reduced by 10-20% every two weeks, but once the dose falls below 10 mg per day, tapering is slowed, usually by 1 mg per month, to minimize the risk of relapse [32]. Given the chronic and recurrent nature of GCA and PMR, adherence to these tapering regimens is essential to balance disease control and minimize steroid-related adverse effects. The use of biologic therapies such as tocilizumab as steroid-sparing agents is an emerging approach that may help in reducing long-term glucocorticoid exposure in refractory or relapsing cases.

For refractory GCA, tocilizumab, an IL-6 receptor inhibitor, has demonstrated significant steroid-sparing effects and improved long-term remission rates, similar to the findings of Castañeda et al. [33]. The study’s findings align with recent trials that support tocilizumab as an effective alternative for patients with frequent relapses or glucocorticoid intolerance [34,35]. The introduction of JAK inhibitors as potential therapeutic options is also being explored, offering hope for patients with severe disease who do not respond to conventional treatments [36]. While corticosteroids remain the cornerstone of treatment for GCA and PMR, careful dose tapering is essential to minimize adverse effects and prevent relapses. Emerging biologic therapies, including IL-6 and JAK inhibitors, offer promising alternatives for patients with refractory disease, potentially improving long-term outcomes and reducing glucocorticoid dependence.

Recent updates in the classification and management guidelines for PMR and GCA have refined diagnostic accuracy and treatment strategies. The ACR and the Chapel Hill Consensus Conference have developed criteria to distinguish GCA from other vasculitides, highlighting key histopathological and clinical markers. Though widely used, the EULAR and ACR provisional classification criteria for PMR have moderate sensitivity (66%) and specificity (81%), reinforcing the need for clinical judgment, inflammatory markers, and imaging studies for accurate diagnosis. Additionally, BSR and EULAR management guidelines now emphasize glucocorticoid tapering protocols, imaging modalities such as PET-CT and ultrasound, and the incorporation of steroid-sparing agents. These advancements underscore the importance of a multidisciplinary, evidence-based approach to optimizing patient care and improving long-term outcomes.

Limitations and future suggestions

While this study provides valuable insights into the clinical features, diagnostic approaches, and treatment outcomes of PMR and GCA, several limitations should be acknowledged. The single-center design may limit the generalizability of the findings to broader populations and different healthcare settings. However, we attempted to minimize selection bias by including patients from urban and rural areas and applying strict inclusion and exclusion criteria to ensure a homogeneous study population. Despite these efforts, the use of consecutive non-probability sampling may still have influenced the representativeness of the study cohort.

While sufficient for assessing early treatment response and relapse rates, the six-month follow-up period limited the evaluation of long-term disease outcomes such as sustained remission, late relapses, and long-term treatment complications. To address this, we implemented standardized follow-up intervals (weekly in the first month and monthly thereafter) to ensure consistent treatment efficacy and adverse effects monitoring. However, future studies with extended follow-up durations must provide further insights into treatment durability and late-onset complications associated with prolonged glucocorticoid or biologic therapy.

One of the major challenges in this study was the lack of universally validated diagnostic criteria for PMR and GCA, requiring reliance on clinical judgment, inflammatory markers (ESR, CRP), and imaging (CDU, MRA) for diagnosis. Since PMR lacks objective disease-specific findings, there remains a risk of misclassification, particularly in differentiating it from other inflammatory and autoimmune conditions. To address this, we applied strict diagnostic protocols, integrating multimodal assessments, but further research is needed to develop standardized diagnostic criteria. Similarly, while CDU was used to detect the "halo sign" in GCA, its role in long-term disease monitoring remains uncertain, particularly for deeper-seated arteries. The potential application of MRA and PET-CT in disease progression tracking requires further validation to determine their feasibility in routine clinical practice.

Given the absence of randomized controlled trials (RCTs) defining optimal treatment strategies, future research should prioritize multicenter RCTs to establish evidence-based protocols for glucocorticoid tapering and steroid-sparing therapies. While tocilizumab was used in glucocorticoid-resistant cases, its long-term effectiveness and safety in PMR and GCA require further study. Additionally, emerging JAK inhibitors are promising treatment alternatives and should be further evaluated in comparative trials.

This study also highlights regional challenges in diagnosis and treatment access, particularly the limited availability of advanced imaging (MRA, PET-CT) and biologic therapies (tocilizumab) in resource-limited settings. While these challenges were acknowledged, they were not systematically analyzed. Future research should examine how healthcare infrastructure limitations impact patient outcomes and explore strategies to enhance early detection and individualized treatment approaches, particularly in settings with limited access to specialized diagnostic and therapeutic options. Addressing these gaps will be critical in optimizing the global early recognition, accurate diagnosis, and effective management of PMR and GCA.

## Conclusions

This study characterized the clinical features, diagnostic approaches, and PMR and GCA treatment outcomes in an elderly population, highlighting key disease identification and management challenges. The results confirmed proximal muscle pain and morning stiffness were the hallmark symptoms of PMR, while headache, scalp tenderness, and jaw claudication were more specific to GCA. Laboratory markers, including elevated CRP and ESR, remained valuable diagnostic tools, with TAB confirming 76% of GCA cases and vascular ultrasound detecting inflammation in 67%. Imaging techniques such as MRA and PET-CT were useful in complex cases, underscoring the importance of multimodal diagnostic strategies to improve early detection and reduce misdiagnosis.

Treatment outcomes demonstrated that glucocorticoids were effective in achieving remission for most patients, but relapses were more common in those with delayed treatment initiation. While biologic therapy with tocilizumab was required in glucocorticoid-resistant cases, it causes higher relapse rates and metabolic side effects. These findings emphasize the need for early intervention, structured steroid tapering, and vigilant monitoring for adverse effects. Future research should focus on long-term steroid-sparing strategies, optimizing biologic therapies, and improving access to advanced imaging techniques, particularly in resource-limited settings, to enhance disease management and patient outcomes.

## Appendices

**Table 5:** Questionnaire for data collection

Section	Questions/Variables Assessed	Response Type
Demographics	Age	(Years)
	Gender	☐ Male ☐ Female
	Place of Residence	☐ Urban ☐ Rural
Clinical History	Disease Onset (Time Since First Symptoms)	(Months)
	Symptom Duration Before Diagnosis	(Months)
	Presence of Systemic Symptoms (Fatigue, Fever, Weight Loss)	☐ Yes ☐ No
Symptoms	Proximal Muscle Pain (PMR)	☐ Yes ☐ No
	Morning Stiffness (>30 min) (PMR)	☐ Yes ☐ No
	Headache (GCA)	☐ Yes ☐ No
	Scalp Tenderness (GCA)	☐ Yes ☐ No
	Jaw Claudication (GCA)	☐ Yes ☐ No
	Visual Disturbances (GCA)	☐ Yes ☐ No
Laboratory Tests	Erythrocyte Sedimentation Rate (ESR)	(mm/hr)
	C-reactive protein (CRP)	(mg/L)
	Complete Blood Count (CBC)	☐ Normal ☐ Abnormal
Imaging and Biopsy	Temporal Artery Biopsy Performed	☐ Yes ☐ No
	Temporal Artery Biopsy Result (GCA Confirmation)	☐ Positive ☐ Negative
	Color Doppler Ultrasonography (Halo Sign Present)	☐ Yes ☐ No
	Magnetic Resonance Angiography (MRA) Needed	☐ Yes ☐ No
Treatment and Follow-Up	Glucocorticoid Therapy Initiation (Dose)	(mg/day)
	Glucocorticoid Therapy Duration	(Months)
	Response to Glucocorticoid Therapy	☐ Complete ☐ Partial ☐ None
	Need for Dose Adjustment in Glucocorticoid Therapy	☐ Yes ☐ No
	Use of Biologic Therapy (Tocilizumab)	☐ Yes ☐ No
	Response to Biologic Therapy	☐ Complete ☐ Partial ☐ None
	Need for Additional Immunosuppressive Therapy	☐ Yes ☐ No
	Development of Other Treatment-Related Complications (Weight Gain, Hypertension, Infections)	☐ Yes ☐ No
	Development of Hyperglycemia	☐ Yes ☐ No
	Development of Osteoporosis	☐ Yes ☐ No
	Treatment Response (Clinical Remission Achieved)	☐ Yes ☐ No
	Relapse After Initial Treatment	☐ Yes ☐ No
	Relapse Frequency	(Times)
	Time to Relapse	(Months)
	Relapse Severity	☐ Mild ☐ Moderate ☐ Severe
	Comparison of Glucocorticoid vs. Biologic Therapy Outcomes	☐ Yes ☐ No
	Long-Term Follow-Up Data (Symptom Recurrence, Side Effects, Functional Improvement)	☐ Yes ☐ No
